# A retrospective analysis of antimicrobial resistance in pathogenic *Escherichia coli* and *Salmonella* spp. isolates from poultry in Uganda

**DOI:** 10.1080/23144599.2021.1926056

**Published:** 2021-05-19

**Authors:** Steven Kakooza, Adrian Muwonge, Esther Nabatta, Wilfred Eneku, Dickson Ndoboli, Eddie Wampande, Damian Munyiirwa, Edrine Kayaga, Maria Agnes Tumwebaze, Mathias Afayoa, Paul Ssajjakambwe, Dickson Stuart Tayebwa, Sayaka Tsuchida, Torahiko Okubo, Kazunari Ushida, Ken’ichi Sakurai, Francis Mutebi

**Affiliations:** a Central Diagnostic Laboratory, College of Veterinary Medicine, Animal Resources and Biosecurity, Makerere University, Kampala, Uganda; b Department of Genetics and Genomics, the Roslin Institute, University of Edinburgh, Edinburgh, Scotland; c Infectious Diseases Institute, College of Health Sciences, Makerere University, Kampala, Uganda; d College of Veterinary Medicine, Animal Resources and Biosecurity, Makerere University, Kampala, Uganda; e Chubu University, Academy of Emerging Sciences, Kasugai, Japan; f Department of Medical Laboratory Science, Faculty of Health Sciences, Hokkaido University Graduate School of Health Sciences, Sapporo, Japan; g Faculty of Life and Environmental Sciences, Department of Animal Sciences, Teikyo University of Science, Tokyo, Japan

**Keywords:** Antimicrobial-resistance, *E. coli*, *Salmonella* spp., poultry, Uganda

## Abstract

There are increasing reports of antimicrobial treatment failures for bacterial diseases of poultry in Uganda. The paucity of data on antimicrobial resistance (AMR) of pathogenic bacteria in Uganda is a major setback to AMR control. This study investigated the occurrence of fowl typhoid, colibacillosis, and AMR in associated pathogens from 2012 to 2018. Laboratory records from the Central Diagnostic Laboratory (CDL), a National Veterinary Diagnostic Facility located at Makerere University, were reviewed. Archived isolates of the causative bacteria for the two diseases were also evaluated for AMR. The frequencies of the two disease conditions, their clinical and necropsy presentations and the demographic data of the diagnostic samples were summarized from the records. Archived bacterial isolates were revived before antimicrobial susceptibility testing. This was done on Mueller Hinton agar using the disk diffusion method, against 16 antimicrobials of medical and veterinary importance according to the Clinical Laboratory Standards Institute guidelines. A total of 697 poultry cases were presented for bacteriological investigations in the review period. Colibacillosis and salmonellosis had prevalence rates of 39.7% (277/697) and 16.2% (113/697), respectively. A total of 63 and 92 isolates of *Escherichia coli* and *Salmonella* spp., respectively, were archived but 43 (68.3%) *E. coli* and 47 (51.1%) *Salmonella* spp. isolates were recovered and evaluated for AMR. Multidrug resistance was more frequent in *E. coli* (38; 88.4%) than salmonellae (25; 53.2%), (*p* < 0.001). The high prevalence of colibacillosis, salmonellosis and the AMR of associated pathogens warrants immediate institution of appropriate disease control measures.

## 1. Introduction

Avian colibacillosis and salmonellosis have been reported to be among the major bacterial diseases hampering poultry production globally including Uganda [1,2]. They are both caused by pathogenic Gram-negative bacteria (*Escherichia coli* and *Salmonella* spp.) belonging to the *Enterobacteriaceae* family [3]. *Escherichia coli* infections may occur either as primary pathogens or as secondary pathogens [4]. The pathogenic strains of *E. coli* in poultry (avian pathogenic *E. coli*; APEC) harbour various virulence genes and may carry antimicrobial resistance factors which influence disease presentation and treatment outcomes [5,6]. On the other hand, *Salmonella* spp. in poultry are often primary pathogens. *Salmonella* Gallinarum and *Salmonella* Pullorum are exclusively pathogenic to avian species whereas non-typhoidal salmonellae including *Salmonella* Enteritidis and *Salmonella* Typhimurium serovars are ubiquitous and cause clinical infections in a wide range of animals including humans. They thus present great poultry health and public health challenges [7,8].

Colibacillosis in poultry usually manifests with respiratory distress, dejection, reduced appetite, poor growth and the swollen head syndrome. Lesions seen at post-mortem include; yolk sac retention, omphalitis, synovitis, arthritis, polyserositis, coligranulomas, enteritis, cellulitis, panophthalmitis, peritonitis and salpingitis [4]. In its acute form, colibacillosis is characterized by septicaemia resulting in death and in its subacute form by pericarditis, air sacculitis and peri-hepatitis [4]. Salmonellosis in poultry manifests with major clinical signs in chicks and poults which include unabsorbed yolk sac, anorexia, diarrhoea, dehydration, weakness and often causes high mortality. In mature fowls, salmonellosis manifests with anorexia, drop in egg production, increased mortality, reduced fertility and hatchability [9]. Some of the common post-mortem lesions associated with salmonellosis include enlarged liver with necrotic foci or bronze greenish tint of the liver, enlarged spleen with whitish spots, enteritis with necrotic lesions in the mucosa, brown coloured lungs and caeca filled with gelatinous, fibrinous or cheese-like exudate. In some cases of colibacillosis and salmonellosis, infected birds may or may not exhibit any clinical signs [3]. This therefore makes post-mortem examination and bacterial culture, key in diagnosis of colibacillosis and salmonellosis [10]. Globally, salmonellosis and colibacillosis have attracted a lot of attention due to their economic significance especially in chicken. However, for Uganda, there are few reports on the occurrence of these two diseases and the antimicrobial profiles of their causative agents. Consequently, there is no prioritization of capacity development for their diagnosis, prevention and control.

The high incidence and prevalence of bacterial diseases have triggered the prolonged and inappropriate use of antimicrobials. Consequently, there is a progressive loss of their effectiveness with the emergence of AMR [11]. In Uganda, a vast diversity of antimicrobials belonging to majority of the existing antimicrobial classes are imported for poultry health and production, moreover as co-formulations [12]. This, coupled with poor coverage of veterinary services and a predominantly small holder-led animal production sector, is a recipe for antimicrobial abuse, hence the emergence of AMR. *Escherichia coli* and *Salmonella* spp. are among the most important poultry pathogens in Uganda [1]. Therefore, they may be frequently exposed to a number of antimicrobials, thus fostering the emergence of AMR [13]. Due to the limited surveillance capacity, AMR has been hardly studied among poultry in Africa and Uganda in particular [14,15]. A few studies conducted in Uganda have investigated AMR among commensals, especially *E. coli* and *Salmonella* spp. in apparently healthy chicken [16]. These studies have reported alarming levels of AMR for a wide range of antimicrobial classes of bacteria. It is also necessary to understand the AMR profiles of pathogenic bacteria recovered from clinical cases to guide treatment and control. This study investigated the burden of colibacillosis and salmonellosis, and AMR amongst *E. coli* and *Salmonella* spp. isolated from clinical cases of poultry presented to the Central Diagnostic Laboratory, Makerere University, Uganda over a period of 7 years.

## 2. Materials and methods

### 2.1. Research study site and design

This was a retrospective study conducted on poultry cases of colibacillosis and salmonellosis diagnosed between January 2012 and December 2018 at the Central Diagnostic Laboratory (CDL), College of Veterinary Medicine, Animal resources and Bio-security, Makerere University, Uganda. According to the Ministry of Agriculture, Animal Industry and Fisheries (MAAIF), animal disease diagnosis supports the broad objective of the Directorate of Animal resources; to support sustainable animal disease and vector control, market-oriented animal production, food quality and safety; for improved food security and household income. The role of the CDL (which was established in 2011) is to support passive animal disease surveillance efforts together with other regional veterinary laboratories and the National Animal Disease Diagnostic and Epidemiology Center (NADDEC), Entebbe. Altogether, these facilities generate data on diseases to inform national strategic planning and animal health policy. According to Byaruhanga et al. [1], CDL receives samples from nearly almost all the country’s regions. However, the majority of the samples come from central Uganda. All cases of colibacillosis and salmonellosis as well as isolates of *Escherichia coli* and *Salmonella* spp. archived and recovered from the clinical cases of poultry presented to the laboratory during this period were included in this investigation.

### 2.2. *Isolation and identification of* E. coli *and* Salmonella spp.

The procedures done at the laboratory’s bacteriology unit involved non-selective and selective inoculation of aseptically harvested samples on blood agar and MacConkey agar, respectively, prior to incubation at 37°C for 24 hrs. A single colony was then further sub-cultured to obtain a pure culture. Colony characteristics of bacteria such as shape, size, surface texture, edge, elevation and colour, Gram’s staining and biochemical tests (lactose fermentation, methyl red, Voges-Proskauer and Indole production tests) were used for identification of bacteria [17].

### 2.3. Bacterial isolates preservation and recovery

From 2012 to 2018, bacterial cultures done on the chicken samples yielded bacteria, which upon identification as *E. coli* and *Salmonella* spp. were emulsified into uniquely labelled cryovials having preservation media; 10% skimmed milk or brain heart infusion broth supplemented with 20% glycerol. In the review period, a total of 155 isolates (63 *E. coli* and 92 *Salmonella* spp.) were archived at −30°C and −80°C. During the study, archived isolates were recovered as per the procedures by Scythes et al. [18]. Briefly, the procedure involved first inoculating 200 µl of the bacteria stocks in 2 ml of brain heart infusion broth (Oxoid, United States of America) and incubation at 37°C for 24 hrs. The broth was then streaked on MacConkey agar (Conda laboratories, Spain) and xylose lysine dextrose agar (Mastgroup, United Kingdom). An additional 24 hrs broth incubation was done for bacteria that did not grow after the first 24hr broth incubation. The two bacterial species colonies were then identified biochemically using methods described by Khan et al. [19] and Sarba et al. [20]. The following biochemical tests were used for identification of *E. coli* and *Salmonella* spp. (indole, methyl red, Voges-Proskauer, hydrogen sulphide production, urease, citrate and lactose fermentation).

### 2.4. Antimicrobial susceptibility testing

Susceptibility testing on the isolates was done on Mueller Hinton agar using the disk agar diffusion method according to the Clinical Laboratory Standards Institute (CLSI) guidelines [21]. A 4 ml suspension of fresh culture colonies was prepared, equivalent to 0.5 McFarland standard. The surface of the agar was evenly swabbed with the suspension and the plates allowed to dry before introducing the antimicrobial discs. The bacteria were subjected to a panel of 16 antimicrobial agents of both human and veterinary relevance. These included penicillins [ampicillin (10 μg), amoxycillin (25 μg)]; beta lactamase inhibitors [amoxycillin/clavulanic acid (30 μg)]; cephalosporins [cefotaxime (30 μg), cefazolin (30 μg), cefoxitin (30 μg), cefuroxime (30 μg), ceftriaxone (30 μg)]; carbapenems; [imipenem (10 μg)]; aminoglycosides [gentamicin (10 μg), neomycin (30 μg)]; tetracyclines [tetracycline (30 μg)]; quinolones [ciprofloxacin (5 μg), nalidixic acid (30 μg)]; potentiated sulphonamides [trimethoprim/sulfamethoxazole (1.25/23.75 μg)]; phenicols [chloramphenicol (30 μg)]. The plates were then incubated at 37°C for 24 hours. For quality control, *E. coli* ATCC 25,922 was used as a reference strain. Upon incubation, the diameter of the zone of inhibition around the disc was measured using a ruler and results interpreted based on CLSI 2018 guideline. Multidrug resistance was defined as resistance to at least three different classes of antimicrobials [22].

### 2.5. Data collection

The laboratory database was accessed to gather information linked to the cases of colibacillosis and salmonellosis, processes of diagnosis and other demographic information of samples. Inclusion criteria; the laboratory receives different diagnostic samples of various species chiefly food animals, wildlife and pets from the East African region. Only common poultry (chicken, geese, ducks, turkeys) samples from Uganda were reviewed. Also, only cases which had well-kept records were reviewed and included in this study.

### 2.6. Statistical analysis

Data analysis was done using Statistical Package for Social Scientists (SPSS) (version 25.0) and R statistical software. Descriptive statistics were used to summarize the data. Data were presented as frequencies and percentages. The prevalence of colibacillosis and salmonellosis was calculated as proportions of the respective cases confirmed in the review period to that of the number of avian species submitted (and meeting the inclusion criteria) in the same period. The prevalence of AMR was calculated as the proportion of the isolates with resistance to the antimicrobials under investigation as a fraction of the number of isolates analysed. On the other hand, the prevalence of multidrug resistance (MDR) was calculated as a proportion of isolates exhibiting resistance to at least three different classes of antimicrobials out of the total number of isolates for the respective species (*E. coli* and *Salmonella* spp.) exhibiting MDR. The corresponding confidence intervals of prevalence were computed as exact binomial 95% confidence intervals using a calculator from https://sample-size.net/confidence-interval-proportion/. The chi-square test was also used to evaluate significant differences between the prevalence of the two diseases (colibacillosis, salmonellosis) and the type of poultry, region of origin and poultry species. Bivariate logistic regression analyses were done to ascertain the impact of variables (independent factors; purpose of commercial chicken, poultry species, region of origin) on the odds of occurrence of the disease outcomes (dependent variables; colibacillosis or salmonellosis) at 95% level of confidence. Where possible, antibacterial resistance rates in different bacteria were compared using the chi-square test with Yate’s continuity correction. Differences at *p* < 0.05 were considered significant. To identify clusters of isolates with similar antibiogram characteristics, resistance and susceptibility were coded as 0 and 1. A data frame was then generated with sample ID, year and the coded antibiogram profile for each of the 16 antibiotics. These were then analysed using Ridom GmBH, Münster, Germany version 2.1. Cluster analysis was used to group samples by similarity of profile and then visualized using unweighted pair group method with arithmetic mean (UPGMA) to give an output of a dendrogram.

### 2.7. Ethical approvals and consent to participate

All farmers consented to inclusion of the samples they presented to the laboratory for future research. Permission to access the Central Diagnostic Laboratory Bacteria Bank and the sample database was obtained from the Laboratory Management Committee. Both the data extraction from the database and the isolates recovery from the bacteria bank were done by the laboratory technologist in the unit of bacteriology as per the laboratory data and biologics safety policies.

## 3. Results

### 3.1. Demographic information of the poultry cases registered at CDL (2012-2018)

Within the seven-year period, a total of 697 poultry cases suspected to be of bacterial causation were submitted to the CDL for diagnosis by bacterial culture methods (Table 1). The cases were mainly from chicken species (692/697, 99.3%) and few from other species (3/697, 0.4%) (geese, turkeys, ducks). The species of two (0.3%) cases was not recorded. From the chicken cases (692), a proportion of 38.0% (263/692) was reared for egg production (layers), 18.5% (128/692) for meat (broilers) and 11.9% (83/692) for both meat and eggs (dual purpose). Most of the cases were submitted from the districts of central Uganda (536/697; 76.9%) with Wakiso (178/536, 33.2%), Mukono (128/536, 23.9%) and Kampala (115/536, 21.5%) districts presenting the biggest numbers of diagnostic samples (Table 1).

**Table 1.** Descriptive information of the poultry cases received for diagnosis by bacterial culture

	Number of cases	Proportion (%)
**Year of submission**		
2012	4	0.6
2013	65	9.3
2014	157	22.5
2015	129	18.5
2016	163	23.4
2017	83	11.9
2018 **Total**	96 **697**	13.8 **100**
**Poultry species**		
Chicken	692	99.28
Duck	1	0.14
Goose	1	0.14
Turkey Data missing **Total**	1 2 **697**	0.14 0.30 **100**
**Nature of samples for bacterial culture**		
Viscera organs	574	82.4
Swabs ^a^	69	9.9
Eggs **Total**	54 **697**	7.7 **100**
**Region of origin of the cases presented**^b^		
Central	536	76.9
Northern	2	0.3
Western Data missing **Total**	19 140 **697**	2.7 20.1 **100**
**Purpose of commercial poultry**		
Commercial Broilers	128	18.4
Commercial Layers	263	37.7
Dual purpose Data missing **Total**	83 223 **697**	11.9 32.0 **100**

a-swab types were nasal, eye, visceral, faecal, joint, cloacal and intestinal and others not-identified, b-Districts of sample origin and corresponding regions included; **Central**: Gomba, Kalangala, Kalungu, Kampala, Kayunga, Luweero, Masaka, Mityana, Mpigi, Mukono, Wakiso; Nakaseke, Nakasongola; **Western**: Hoima, Kanungu, Kasese, Kiryandongo, Kiruhura, Masindi, Mbarara, Ntungamo and **Northern**: Gulu

At the laboratory level, microbial culture was mainly from visceral organs (574/697,82.4%) including the liver, heart, lungs, spleen, air sacs and the yolk sacs. Others were swabs (69/697,9.9%) mainly nasal (n = 2), eyes (n = 8), visceral (n = 1), faecal (n = 1), joint (n = 1), cloacal (n = 11), intestinal (n = 1), unidentified swab type (n = 44) and eggs (54/697,7.7%) (Table 1). Swabs were taken from either freshly dead or sick birds at post-mortem performed either at the laboratory or from the farms. During the review period, CDL received poultry samples from 16.4% (22/134) of the districts in Uganda.

### 3.2. Clinical signs and necropsy gross lesions in the affected flocks and their birds

Irrespective of the disease, the records showed that affected flocks and their birds submitted were seen to be presenting with various signs. Majority of the cases originated from flocks characterized by over 10% mortality rates (234, 33.6%), weak birds (95, 13.6%), diarrhoea (71, 10.2%) (brown, white, green, yellow, blood stained), dullness (47,6.7%), emaciation (36, 5.2%), drop in production (34, 4.9%), coughing (33, 4.7%), egg hatching failures (28, 4.0%), respiratory distress (28, 4.0%), rhinorrhea (25, 3.6%) and reduced appetite (23, 3.3%). Other signs seen included; paralysis (15, 2.2%), swollen joints (4, 0.6%), lameness (7, 1.0%), respiratory rales (10, 1.4%), spreading out of wings (1, 0.1%), stunted growth (8, 1.1%), ruffled feathers (12, 1.7%), twisted neck (9, 1.3%), droopy wings (9, 1.3%), recumbency (5, 0.7%), lameness (7, 1.0%) and swollen heads (2, 0.3%).

**Figure 1.**
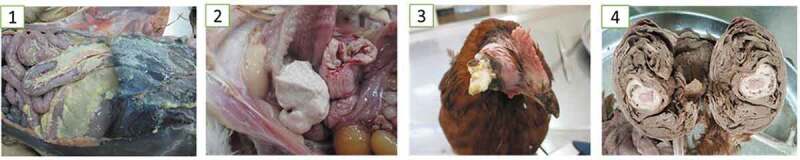
Major morphological presentations of the detected cases of colibacillosis. 1. Diffuse acute fibrinous peritonitis, perihepatitis and air sacculitis; 2. Localized Chronic fibrinous salpingitis; 3. Bilateral panophthalmitis and 4. Severe obliterative chronic salpingitis

**Figure 2.**
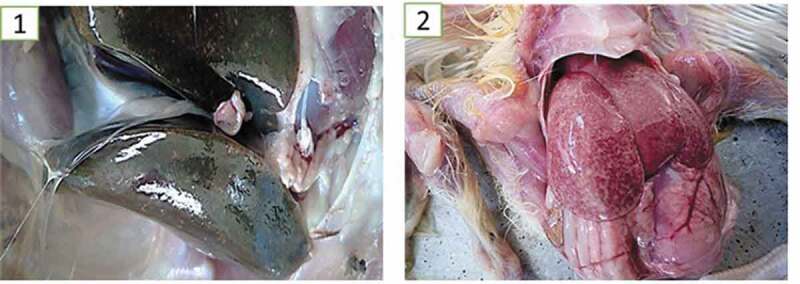
Common morphological presentations of salmonellosis. 1. Greenish discolouration in the liver with multifocal necrotic foci in the parenchyma; 2. Multifocal to diffuse hepatic necrotic foci and hepatic congestion

At necropsy, the common lesions associated with *E. coli* infections were mainly acute focal to diffuse fibrinous air sacculitis, peritonitis, perihepatitis, pericarditis, panophthalmia and chronic fibrinous salpingitis (Figure 1) whereas the common lesion seen in salmonellosis cases was mainly severe diffuse hepatic necrosis with a greenish bronze discolouration (Figure 2).

### 3.3. Prevalence of colibacillosis

Out of the 697 cases presented to the laboratory, the overall prevalence of colibacillosis was 39.7% (n = 277; CI = 36.1–43.5) of which 2.7% (n = 19; CI = 1.7–4.2) of the cases were diagnosed as co-infections with salmonellosis. All the cases of colibacillosis were detected in chicken. The prevalence in broilers, layers and dual purpose was 56.3% (72/128; CI = 47.2–65.0), 44.5% (117/263; CI = 38.4–50.7) and 36.1% (30/83; CI = 25.9–47.4), respectively. There was a significant difference in the prevalence of colibacillosis amongst the various types of commercial chicken (*X^2^ *= 8.89, df = 2, *p* = 0.012). Apparently, the risk of contraction of colibacillosis was lowered in dual purpose (OR = 0.44, CI = 0.25–0.78) and layers (OR = 0.62, CI = 0.41–0.95). Of the 277 confirmed cases, the central region contributed the major proportion (75.8%; n = 210; CI = 70.3–80.7) and few cases came from other regions. Most of the confirmed cases came from Wakiso (26.4%,73/277; CI = 21.3–32.0), followed by Mukono (19.9%, 55/277; CI = 15.3–25.1), and then Kampala (11.2%, 31/277; CI = 7.7–15.5). The occurrence of cases of colibacillosis was not influenced by the region the cases came from (*X^2^ *= 2.70, df = 2, p = 0.259) (Table 2).

**Table 2.** Prevalence of colibacillosis and salmonellosis in commercial poultry, regions of sample origin, and poultry species

Variable	Number of Submissions	Diagnosis by bacterial culture	
		Colibacillosis (x, %) [*p*-value; CI; OR]	Salmonellosis (y, %) [*p*-value; CI; OR]
Purpose of commercial poultry*			
Broiler	128	72 (56.3) [a; a; 1.00]	27 (21.1) [0.022; 1.11–3.39; 1.93]
Dual	83	30 (36.2) [0.005; 0.25–0.78; 0.44]	21 (25.3) [0.005; 1.32–4.54; 2.45]
Layers	263	117 (44.5) [0.029; 0.41–0.95]	32 (12.2) [a; a; 1.00]
Data missing	223	58 (26.0) [a]	33 (14.8) [a]
Total	**697**	**277 (39.7**) [a]	**113 (16.2)** [a]
**Region of origin**			
Central	536	210 (39.2) [a; a; 1.00]	94 (17.5) [a; a;1.00]
Northern	2	0 (0.0) [0.999; a; a]	0 (0.0) [0.999; a; a]
Western	19	10 (52.6) [0.244; 0.69–4.316; 1.73]	4 (21.1) [0.693; 0.41–3.86; 1.25]
Data missing	140	57 (40.7) [a]	15 (10.7) [a]
**Total**	**697**	**277 (39.7)** [a]	**113 (16.2)** [a]
**Poultry species**			
Chicken	692	277 (40.0) [a; a; 1.00]	113 (16.3) [a; a; 1.00]
Duck	1	0 (0.0) [1.000; a; a]	0 (0.0) [1.000; a; a]
Goose	1	0 (0.0) [1.000; a; a]	0 (0.0) [1.000; a; a]
Turkey	1	0 (0.0) [1.000; a; a]	0 (0.0) [1.000; a; a]
Data missing	2	0 (0.0) [a]	0 (0.0) [a]
**Total**	**697**	**277 (39.7)** [a]	**113 (16.2)** [a]

CI – Confidence interval, OR – Odds Ratio, a – no computation, *significant predictor for both colibacillosis and salmonellosis.

### 3.4. Prevalence of salmonellosis

The overall prevalence of salmonellosis was 16.2% (n = 113; CI = 13.6–19.2) with 2.7% (n = 19; CI = 1.7–4.2) of the cases being diagnosed as co-infections with colibacillosis. All cases of salmonellosis were diagnosed in chicken. The prevalence in broilers, layers and dual-purpose was 21.1% (27/128; CI = 14.4–29.2), 12.2% (32/263; CI = 8.5–16.7) and 25.3% (21/83; CI = 16.4–36.0), respectively. There was a significant difference in the prevalence of salmonellosis among commercial chicken of different purposes (*X^2^ *= 9.98, df = 2, *p* = 0.007). The risk of getting salmonellosis was increased in the dual purpose (OR = 2.45, CI = 1.32–4.54) and broiler birds (OR = 1.93, CI = 1.11–3.39) when compared to layers. Most cases of salmonellosis were from central Uganda (82.3%; 93/113). Among districts, Wakiso presented the biggest number of cases (51/113; 45.1%), followed by Kampala (13/113; 11.5%) and Mukono (13/113; 11.5%). There was no significant difference in the prevalence of salmonellosis among regions (*X^2^ *= 0.59, df = 2, *p* = 0.746) (Table 2).

### 3.5. Bacterial investigations on antimicrobial resistance

Out of a total of 155 (*E. coli*, n = 63; *Salmonella* spp., n = 92) isolates archived in the laboratory for the period 2012–2018, only 90 (58.1%) isolates were recovered. The recovery rates for *E. coli* and salmonellae were 68.3% (43/63) and 51.1% (47/92), respectively.;

Overall, out of the 90 isolates, highest resistance was majorly observed with Amoxycillin (65.6%, n = 59), followed by cefazolin (63.3%, n = 57), nalidixic acid (54.4%, n = 49), ciprofloxacin (44.4%, n = 40), tetracycline (42.2%, n = 38), neomycin (36.7%, n = 33) and trimethoprim sulphamethoxazole (35.6%, n = 32). Minimal resistances were observed with cefoxitin (1.1%, n = 1), ceftriaxone (6.7%, n = 6), amoxycillin clavulanic acid (12.2%, n = 11) and imipenem (13.3%, n = 12). Multidrug resistance was shown by 70% (n = 63) of the isolates.

Out of the 43 isolates of *E. coli*, highest resistance was majorly observed with Amoxycillin (88.4%, n = 38), followed by tetracycline (86.0%, n = 37), cefazolin (83.7%, n = 36), trimethoprim sulphamethoxazole (69.8%, n = 30). Minimal resistances were observed with cefoxitin (0.0%), ceftriaxone (7.0%, n = 3), cefuroxime (7.0%, n = 3) and imipenem (11.6%, n = 5). Multidrug resistance was shown by 88.4% (n = 38) of the isolates.

Out of the 47 isolates of salmonellae, highest resistance was majorly observed with ciprofloxacin (55.3%, n = 26), followed by amoxycillin (44.7%, n = 21), nalidixic acid (44.7%, n = 21), cefazolin (42.6%, n = 20), neomycin (29.8%, n = 14). Minimal resistances were observed with chloramphenicol (0.0%), ceftriaxone (6.4%, n = 3), tetracycline (2.1%, n = 1). Multidrug resistance was shown by 53.2% (n = 25) of the *Salmonella* isolates.

Pan resistance relating to the 16 antibiotics was not found. Antibiotic resistance to ampicillin, amoxycillin, tetracycline, chloramphenicol and trimethoprim sulphamethoxazole was significantly more common in *E. coli* isolates (*p* < 0.05) whereas resistance to amoxycillin clavulanic acid was significantly more common in *Salmonella* spp. (*p* < 0.05) (Table 3).

**Table 3.** Comparison of AMR in pathogenic *E. coli* and *Salmonella* spp

Antibiotic resistance type	Family	Number of isolates (%; CI)		*p*-value
		*E. coli* (n = 43)	*Salmonella* spp. *(n = 47)*	
Ampicillin	β-lactams	24 (55.8; 39.9–70.9)	11 (23.4;12.3–38.0)	0.003*
Amoxycillin		38 (88.4;74.9–96.1)	21 (44.7;30.2–59.9)	< 0.001*
Amoxycillin clavulanic acid	Modified penicillins	1 (2.3;0.0–12.3)	10 (21.3;10.7–35.7)	0.016*
Ciprofloxacin	Quinolones	14 (32.6; 19.1–48.5)	26 (55.3;40.1–69.8)	0.05
Nalidixic acid		28 (65.1;49.1–79.0)	21 (44.7;30.2–59.9)	0.083
Gentamicin	Aminoglycosides	11 (25.6;13.5–41.2)	8 (17.0;7.7–30.8)	0.462
Neomycin		19 (44.2;29.1–60.1)	14 (29.8;17.3–44.9)	0.231
Cefazolin	Cephalosporins (1^st^, 2^nd^, 3^rd^)	36 (83.7;69.3–93.2)	20 (42.5;20.3–57.8)	< 0.001*
Cefoxitin		0 (0.0;0.0–8.2)	1 (2.1;0.0–11.3)	1.000
Cefuroxime		3 (7.0;1.5–19.1)	9 (19.1;9.2–33.3)	0.166
Cefotaxime		9 (20.9;10.0–36.0)	13 (27.7;15.6–42.6)	0.620
Ceftriaxone		3 (7.0;1.5–19.1)	3 (6.4; 1.3–17.5)	1.000
Imipenem	Carbapenems	5 (11.6;3.9–25.1)	7 (14.9;6.2–28.3)	0.885
Tetracycline	Tetracyclines	37 (86.0;72.1–94.7)	1 (2.1;0.0–11.3)	< 0.001*
Chloramphenicol	Phenicols	15 (34.9;21.0–51.0)	0 (0.0;0.0–7.6)	< 0.001*
Trimethoprim sulphamethoxazole	Potentiated sulphonamides	30 (69.8;53.9–82.8)	2 (4.3;0.0–14.5)	< 0.001*
MDR (≥ 3 antimicrobials)		38 (88.4;74.9–96.1)	25 (53.2;38.1–67.9)	<0.001*

Statistical analysis: *X*^2^ test with Yate’s continuity correction; CI-Confidence Interval.

* Statistically significant.

A total of 63 strains (38 *E. coli* and 25 salmonellae) exhibited multidrug resistance (MDR). The prevalence of MDR was higher in *E. coli* (88.4%) compared to salmonellae (53.2%) (*p* < 0.05) (Table 3). The MDR classes were profiled and the maximum number of antimicrobials against which an isolate was resistant was 13 belonging to quinolones, aminoglycosides, phenicols, cephalosporins, tetracyclines, penicillins and potentiated sulphonamides demonstrated by one *E. coli* isolate. However, the most widespread MDR class was resistance to 7 drugs (Table 4).

**Table 4.** Profiling of multidrug resistance (MDR) in *E. coli* and *Salmonella* spp

MDR* Categories	Number of isolates	Number of isolates, n (%)	
		*E. coli* (n = 38)	*Salmonella* spp. (n = 25)
Three	5	0 (0.0)	5 (20.0)
Four	6	3 (7.9)	3 (12.0)
Five	10	8 (21.1)	2 (8.0)
Six	9	6 (15.8)	3 (12.0)
Seven	15	4 (10.5)	11 (44.0)
Eight	10	10 (26.3)	0 (0.0)
Nine	1	1 (2.6)	0 (0.0)
Ten	4	3 (7.9)	1 (4.0)
Eleven	2	2 (5.3)	0 (0.0)
Thirteen	1	1 (2.6)	0 (0.0)

*Characterization of multidrug resistance (MDR) by an isolate was defined as resistance to at least one agent in three or more different families of antimicrobials

### 3.6. Clustering of isolates resistance profiles

**Figure 3.**
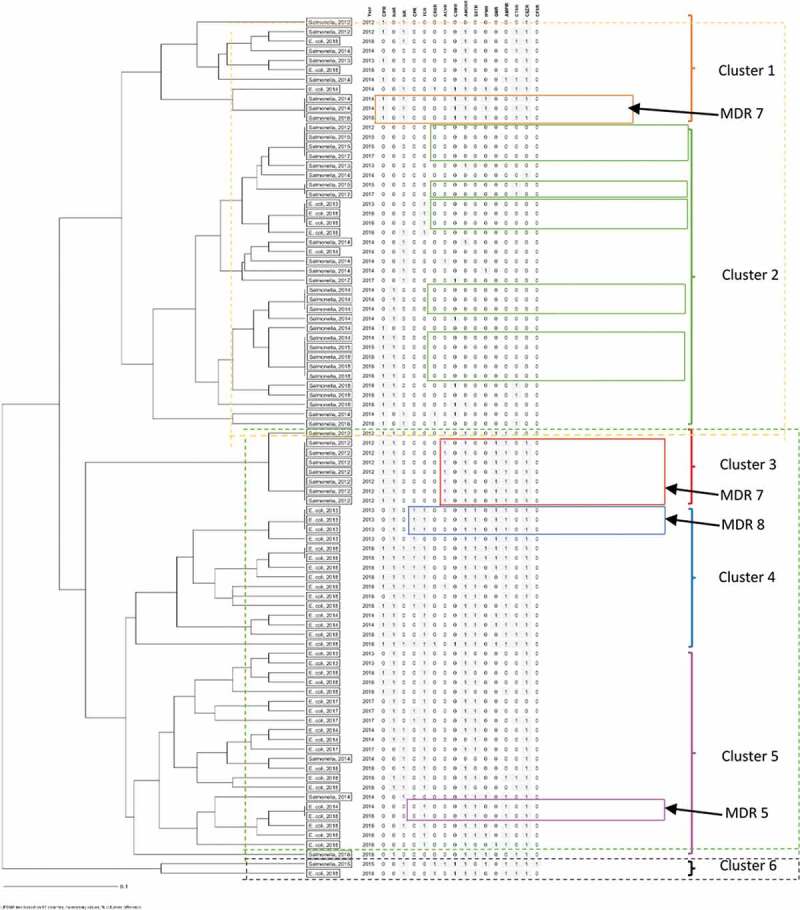
The UPGMA dendrogram shows the relationships of *E. coli* and *Salmonella* spp. isolates (N = 90) based on phenotypic resistance to 16 antibiotics. Resistance and no resistance were coded as 0 and 1 respectively and using Ridom GmBH, Münster, Germany isolates were clustered by similarity of profile on the 16 antibiotics (antibiogram). We identified six major clusters spanning the seven-year period. Within these clusters, we observe several multi-resistant isolates marked with an arrow

Clusters 1 and 2 contain identical salmonellae isolates resistant to several antibiotics (MDR), which surprisingly span the entire temporal scale of the study. This suggests that these are likely endemic in the study area. Cluster 3 is exclusively made of salmonellae isolates from 2012, on the other hand, cluster four consists of *E. coli* recovered across temporal scales. It is noteworthy that identical isolates were also observed in the same years (Figure 3).

## 4. Discussion

This study emphasizes the role of accurate diagnosis in prevention and control of common poultry diseases on farms. If fresh samples are presented and standard aseptic bacteriological procedures are done, bacterial culture remains a gold standard diagnostic test for confirmation of colibacillosis and salmonellosis in poultry [4]. The burden of colibacillosis and salmonellosis in the referred cases was 39.7% and 16.2%, respectively. The prevalence rates obtained imply the endemicity of the two diseases in Uganda. The burden of colibacillosis was higher than salmonellosis, which was in agreement with another study by Byaruhanga et al. [1]. This may point to the poor implementation of infection prevention and control measures focusing on biosecurity and hygienic practices among the poultry units from which the samples were drawn.

Many strains of *E. coli* and salmonellae hosted by birds are pathogenic to humans [4]. Thus, the occurrence of these diseases in poultry, a popular food animal, questions the safety of poultry products especially in developing countries like Uganda with liberal food safety systems. The prevalence of colibacillosis was highest in broilers (56.3%) and this was slightly greater than 53.4% obtained by Ibrahim et al. [23] and much lower than 88.2% by El Sukhon et al. [24]. Elsewhere, the prevalence has been reported to be in the range of 52.26% to 86.7% [25]. The varying prevalence rates could be as a result of sample size variations used by the researchers. The probable risk of occurrence of colibacillosis was lessened with layers and dual-purpose birds. Unlike layers and dual-purpose birds, the high feed intake by broilers arouses much shedding of faeces, thus increasing the risk of their environmental contamination, a predisposing factor to disease. Also, broiler production systems tend to have higher stocking densities compared to layers and dual-purpose chicken. This presents challenges for environmental management aimed at minimizing the risk of *E. coli* infections.

Salmonellae are endemic in poultry [26] with the majority of reports focusing on layers and broilers neglecting the dual-purpose breeds. The analysis shows that the prevalence of salmonellosis was highest in the dual-purpose birds. The dual purpose and broiler birds were at an increased risk of contraction of salmonellosis. This could be as a result of higher rate of salmonellae contamination on broiler farms due to poor management practices right from the hatcheries and then to the raising houses as noted by Ahmed et al. [26]. Also, in Uganda, dual purpose chicken multiplication is majorly in the hands of small holder farmers and emerging uncertified hatcheries that hardly pay attention to critical zoosanitary practices aimed at disease control.

Over the years, a number of antimicrobial resistance phenotypes have emerged. These present a serious hazard to human and animal health [27]. The results highlight the AMR patterns of the major pathogens of poultry in Uganda. This provides critical insights to poultry veterinary practitioners for the treatment of colibacillosis and salmonellosis outbreaks on farms.

Penicillins, tetracyclines and potentiated sulphonamides are some of the old antibiotics which have existed on the Uganda market for decades, thus in the long run acquiring resistance. Tetracyclines have also been recorded in Uganda and other countries to be widely used in treating bacterial diseases and growth promotion in the animal industry [14,28]. It is therefore not surprising that there is widespread resistance of pathogens against them. The continuous exposure to antimicrobials induces a selection pressure in the bacteria [29] such as commensal *E. coli*. These with time develop into MDR strains which can also be pathogenic at a future time. This explains why most of the *E. coli* isolates had AMR to amoxycillin (88.4%), tetracycline (86.0%), cefazolin (83.7%), trimethoprim sulphamethoxazole (69.8%), nalidixic acid (65.1%) and ampicillin (55.8%).

In comparison with *E*. coli, a bigger gap globally exists in the documentation of AMR in *Salmonella* spp. clinical isolates from poultry. In this study, quinolone and fluoroquinolone resistances were the most prevalent in *Salmonella* spp. The isolates were chiefly resistant to ciprofloxacin (55.3%), nalidixic acid (44.7%), amoxycillin (44.7%), cefazolin (42.6%), neomycin (29.8%) and cefotaxime (27.7%). Coincidentally, many of these drugs fall under the most widely used antimicrobial families for salmonellosis treatment in poultry in Uganda (Mutebi, personal observation). High quinolone resistances were also reported by other researchers [30–32] but our findings were different from those of Gong et al. [33] whose isolates revealed the peak rates of resistance to trimethoprim, streptomycin, tetracycline and sulfamethoxazole. On the other hand, lower resistance occurrence was observed for gentamicin, ciprofloxacin, chloramphenicol, kanamycin and cefotaxime. This could probably be due to the variations in the biovars of the enterica group used in the studies and also the difference in the regimen of antibiotic usage where some of the drugs had not yet been extensively used.

The study revealed that resistance was less dominant in the newer quinolones tested in both populations. Nalidixic acid, an old-time quinolone, showed higher resistance rates compared to ciprofloxacin. This was also seen in similar studies [17,34,35]. In the aminoglycoside family, neomycin resistance was higher than gentamicin resistance for both bacterial species. This was also reported in the studies by Chansiripornchai et al. [36] for *E. coli* and Taddele et al. [31] for salmonellae. The use of gentamicin in poultry is limited and the Ugandan Veterinary register has only one product which can be used in poultry, which in actual sense was recently introduced [12].

Cephalosporins, carbapenems and phenicols usage in Ugandan poultry are minimal [14]. Therefore, it is worrying that the study reports mild resistances to the antimicrobials falling in the three classes. However, there exists a possibility of resistance emerging when farmers illegally use human drugs especially if there seems to be no response with the existing veterinary drugs. This has been documented in some parts of the world where human drug residues have been found in animal products [37]. This may be one of the drivers for the emerging resistances. Low *E. coli* resistances to amoxycillin clavulanic acid give optimism for taming the colonization of extended spectrum beta-lactamases (ESBLs) producing strains (mostly cefotaxime and cephalosporin resistant bacteria) in poultry and crossing over of such strains to humans. This could be due to its low application in the treatment of poultry bacterial infections. Some studies [38,39] reported a prevalence range of 73% to 100% of multi-drug resistant *E. coli*, which is in line with the findings of this study (88.4%). However, findings by another study [33] reported a 76.6% MDR prevalence in *Salmonella* spp. which is higher than the prevalence (53.2%) reported by this study. *Escherichia coli* is more ubiquitous than *Salmonella* spp. [40]. Thus, increasing its chances of acquiring AMR, which also exhibited significantly high resistance in more drug classes tested with it. Although relatively low, *Salmonella* spp. displayed resistance to the beta lactam antibiotic family (penicillins, cephalosporins and carbapenems) which explains its high resistance rate to amoxycillin clavulanic acid, a combination drug with beta lactamase inhibiting properties. It is also surprising that unlike *E. coli*; low levels of tetracycline-resistant salmonellae were observed despite the current widespread country-wide tetracycline resistance. This was also reported in a study by Penha [32]. Other studies [41,42] reported that the carriage of tetracycline resistance genes is more predominant in *E. coli* compared to salmonella*e*. It is also reported that the *E. coli* genome is more flexible than the *Salmonella* spp. genome in the gene transfer [43] thus its DNA can be modified through genomic expansion, deletion, and rearrangement, thus yielding more pathogenic *E. coli* strains [43].

From the cluster analysis, identical isolates that span temporal scales suggest endemic prevalence of resistance to certain antibiotics in the antibiogram. This characteristic of endemic resistance was more common with salmonellae compared to *E. coli*. It was observed that the highest number of antibiotics to which isolates were resistant was eight (MDR 8) and there were no pan susceptible isolates.

Only 90 isolates out of over 600 samples were successfully stored and analysed because of resource constraints (majorly biobank freezer space and management). In particular, unreliable electricity supplies to the laboratory contributed immensely to the loss of the archived bacterial isolates. Nonetheless, the results provide preliminary insights into AMR in the poultry sector. The absence of pan susceptible isolates and the high level of MDR among *E. coli* and salmonellae is alarming.

This study gives a baseline picture of AMR in clinical cases of *E. coli* and *Salmonella* spp. in poultry in Uganda. However, there is a need to study the emergence and transmission drivers involved which may be host (demographics such as sex, breed, flock size), agent (AMR genes, drug target mutations) and environment (antimicrobial consumption or usage practices) related. Also, the study had limitations since it was a retrospective study, a number of isolates were lost during preservation thus affecting the sample size. At the animal-human interface, the two pathogens are zoonotic, thus the chances of transmission of antibacterial resistant bacteria (ARB) are high and these dynamics ought to be studied. Transmission of ARB can occur for example through consumption of contaminated poultry and poultry products. Also, there are occupational risks posed on personnel in direct contact with diseased flocks such as farmworkers and slaughterhouse attendants.

## 5. Conclusion

The study demonstrates that colibacillosis and salmonellosis are endemic in Ugandan poultry especially amongst broiler birds. Their causative agents are also highly resistant to the most common antibacterial agents currently in use. It is thus prudent that infection prevention and control measures such as vaccination (especially for colibacillosis, fowl typhoid and pullorum disease), good farm biosecurity practices, sensible antimicrobial use, management and hygiene in flocks and at hatcheries be instituted. Comparatively, *E. coli* had higher resistance to a number of antimicrobials than salmonellae. The emergence of resistance in lowly consumed antimicrobial classes in animals but vastly used in humans calls for the development of a nationwide surveillance programme for monitoring of the World Health Organization AMR priority pathogens. Further studies on the molecular mechanisms to back up the phenotypic AMR data are also encouraged.

## Data Availability

The datasets used and/or analysed during the current study are available from the corresponding author on reasonable request.
